# Alcohol, Smoking and Substance Involvement Screening Test validity in bipolar and psychotic disorders

**DOI:** 10.4102/sajpsychiatry.v29i0.2109

**Published:** 2023-12-21

**Authors:** Rosalind J. Adlard, Tessa Roos, Henk Temmingh

**Affiliations:** 1 Department of Psychiatry and Mental Health, Faculty of Health Sciences, University of Cape Town, Cape Town, South Africa

**Keywords:** substance use disorders, psychotic disorders, bipolar disorders, validity, screening test

## Abstract

**Background:**

Patients with multi-episode bipolar and psychotic disorders have a high prevalence of substance use disorders, with negative consequences. A brief, easily administered screening test such as the Alcohol, Smoking and Substance Involvement Screening Test (ASSIST) is needed to identify those at risk in order to intervene appropriately. However, the ASSIST has not yet been validated in this population.

**Aim:**

This article aims to determine the validity and reliability of the ASSIST in detecting substance use disorders in patients with multi-episode bipolar and psychotic disorders.

**Setting:**

Western Cape Province, South Africa.

**Methods:**

The Structured Clinical Interview for the Diagnostic and Statistical Manual of Mental Health Disorders, 4th Edition (DSM-IV) Axis I Disorders (SCID-I) was used as the gold standard for detecting substance abuse and dependence. Cronbach’s alpha was used to determine the internal consistency of the ASSIST, and receiver operating characteristic analysis was used to evaluate its screening properties. Optimal cut off scores were calculated to maximise sensitivity and specificity.

**Results:**

A total substance involvement lifetime score of ≥13 was found to have optimal sensitivity and specificity of just over 74%. The optimal cutoff score for alcohol was ≥4 and for cannabis, methamphetamine, and ‘other drugs’ was ≥3. The area under the curve was 0.7 or above for both the total and specific substance involvement scores.

**Conclusion:**

The ASSIST is a psychometrically sound screening test for substance use disorders in patients with multi-episode bipolar and psychotic disorders.

**Contribution:**

This is the first study to validate the ASSIST in this population.

## Introduction

South Africa has a lifetime prevalence of 13.4% of substance use in the general population, while the prevalence of mental illness as defined by a Diagnostic and Statistical Manual of Mental Disorders, Fourth Edition, Text Revision (DSM-IV-TR) diagnosis is 30.3%.^1^ Substance use disorders (SUD), which the DSM-IV-TR classifies into substance abuse disorders and substance dependence disorders,^2^ occur at a higher rate (odds ratio [OR]: 2.7) in those with a diagnosed mental illness compared to those without one.^3^ Substance use disorders are often under-detected in those with mental illness,^4^ which is problematic as the co-occurrence of these disorders worsens the prognosis of each disorder.^5^ The comorbidity of SUDs with mental illness is associated with more severe illness and a worse illness course.^5^ This in turn is associated with health problems including longer duration of untreated psychosis^6^; increased risk of relapse; more psychological distress; poorer medication compliance; higher rates of utilisation of health services; impaired psychosocial functioning; and increased rates of institutionalisation, violence, suicide, forensic problems.^4,7^ Screening for substance use is thus recommended in order to detect these disorders and enable appropriate and timeous interventions to take place.^8^ However, many tests are too cumbersome to be appropriate for overburdened health services, and few have been validated in developing countries.^9^

The Alcohol, Smoking and Substance Involvement Screening Test (ASSIST) is a substance use screening tool developed by the World Health Organization (WHO).^10^ To date, the ASSIST has been validated in the primary care context,^11^ in emergency settings,^12^ and in patients with first episode psychosis.^13^ However, it has not yet been validated in the context of multi-episode, established bipolar and psychotic disorders.

Given the high rate of SUDs in patients with recurrent bipolar and psychotic disorders, there is a need for a brief screening test such as the ASSIST to be validated in this population, thus enabling health care workers to identify SUD and to intervene appropriately where needed. The study aim is thus to determine the validity of the ASSIST in patients with multi-episode bipolar and psychotic disorders, as well as in controls without these illnesses.

## Research methods and design

### Study design

This study is a secondary data analysis of an existing database. The original database has been derived from a completed case-control study, investigating electroencephalogram (EEG) delta/alpha frequency activity in patients with a diagnosis of psychotic disorder, compared to controls without a psychotic illness.^14^

### Study setting and sample

In the original study, participants were recruited using word of mouth methods, referral from clinicians, and media advertisements. Clinically stable outpatients from the Western Cape province of South Africa were selected as cases. Controls were from similar socio-economic backgrounds as the participants, but without a history or diagnosis of psychosis or bipolar disorder (BMD). Cases were required to have a diagnosis of schizophrenia (SZP), BMD with psychosis, or methamphetamine induced psychotic disorder (MPD), diagnosed as per the Structured Clinical Interview for DSM-IV-TR Axis I Disorders (SCID-I). They were required to be between 19 and 40 years of age and to be fluent in English. Exclusion criteria included any general medical conditions that required chronic treatment; history of learning disability; history of major brain injury or surgery; history of cardiovascular insult; individual or family history of epilepsy; and any medical implants or metal within their bodies. Females who were pregnant or lactating were excluded; and patients with a diagnosis of BMD or SZP were excluded if their disorder was deemed to be substance induced. However, co-morbid substance use *per se* was not excluded (including for the non-psychotic control group). The patients with MPD were required not to have evidence for another primary psychotic disorder.^14^

For the current analysis, patients with bipolar type II disorder who may have had psychotic symptoms as part of a major depressive episode, but not part of hypomania, were included. All participants in the dataset who completed the ASSIST were included. The sample size of this study was determined and limited by the data available in the parent study.

### Measures and data collection

On the day of assessment all participants completed both the SCID-I and ASSIST. The SCID-I is a semi-structured interview, designed to detect Axis I disorders as defined in the DSM-IV. It is commonly used in research settings and its reliability has been demonstrated.^15^ The principle bipolar or psychotic disorder diagnosis as well as the DSM-IV SUD diagnosis (either substance abuse or substance dependence) was determined by the SCID-I, utilising modules A, B, C, D, and E. It uses a combination of closed and open-ended questions and is administered by trained interviewers. It takes an average of 3 h to administer.

The ASSIST version 3.0 is a self-report screening tool which was developed by the WHO to screen for psychoactive substance use and related problems in primary care patients.^10^ It consists of eight items that measure recent (i.e. past 3 months) and lifetime use of 10 substances. The ASSIST can be administered in 5–10 min, and is conducted in an empathic, non-judgmental manner, using open ended questions. The ASSIST begins with a broad question regarding lifetime substance use; it goes on to cover frequency of use, cravings, frequency of substance related problems (health, social, legal, or financial) and effects on role responsibilities, in the last 3 months. Regarding lifetime use, it asks whether others are concerned, past attempts to cut down on substance use, and any intravenous drug use.^10^ Each question on the instrument is differentially weighted with a Likert-type scale. A total substance involvement score (TSI) is obtained by summation of scores on Q1 through to Q8 that measure both recent and lifetime substance use across all substances used. It can also assess past 3-month substance use only (TSI-3-month; the sum of Q2–Q5). In addition, Specific Substance Involvement (SSI) scores can be calculated separately for each substance by summation of score across items Q2 to Q7.

### Data analysis

The relevant variables, notably the ASSIST scores and the DSM-IV substance use diagnoses, were extracted from the data and tabulated. The SCID-I was used as the gold standard to determine the presence or absence of a DSM-IV SUD – either substance abuse or substance dependence. Cronbach’s alpha was used to determine the internal consistency, or reliability, of the ASSIST. The discriminant validity of the ASSIST was determined by comparing the TSI and SSI scores on the ASSIST obtained from patients with a SUD and to those without a SUD. Receiver Operating Characteristic (ROC) curves were used to evaluate the screening properties of the ASSIST. Optimal cut off scores were calculated to maximise sensitivity and specificity. The sensitivity, specificity, and positive and NPVs were calculated. We determined the normality of the data using histograms and the Shapiro Wilk’s test for normality. Skewed data were normalised using logarithmic transformations where appropriate. Normal data were analysed using Student’s t-test and Pearson’s correlation coefficient. For non-normal data, Wilcoxon-rank sum test or Spearman’s rank correlation coefficient were used. Categorical data were analysed using Chi-square test with Fisher’s exact test where appropriate. For comparisons across three groups on the ASSIST TSI and SSI scores (no SUD, abuse, and dependence), Kruskal–Wallis analysis of variance (ANOVA), with a Bonferroni correction for post-hoc pairwise comparisons, was used. Following the determination of optimal cut-points on the TSI scores, a logistic regression model was constructed to determine the unadjusted odds of having a SUD after testing positive (scoring at or above the cut-score) and the adjusted odds after accounting for covariates (sex, education level, and diagnostic group). Two-tailed tests were used throughout, with *p*-values <0.05 considered as statistically significant. Stata version 16 for Windows was used to analyse data.

### Ethical considerations

The University of Cape Town’s Human Research Ethics Committee (HREC) granted approval for the study (HREC 833/2019). As this is a secondary analysis of an existing dataset, the ethical considerations and risks were considered minimal.

## Results

### Sample characteristics

Of the 124 participants, 58 (46.8%) had a SUD diagnosis on the SCID-I, and 66 (53.2%) did not. Alcohol and methamphetamine were the most commonly abused substances, followed by cannabis and ‘other drugs’ (which includes methaqualone, cocaine, hallucinogens, opioids, and inhalants). A SUD diagnosis was significantly associated with gender (higher in males), having less than 12 years of education, and a diagnosis of either SZP, BMD, or MPD or non-psychotic control status (Table 1).

**TABLE 1:** Sociodemographic and clinical characteristics of study participants.

Sociodemographic and clinical variables	Total *N* = 124		SUD absent *N* = 66		SUD present *N* = 58		Statistical test	*P*
	*n*	%	*n*	%	*n*	%		
**Age: mean (s.d.)**	28.2	5.4	28.1	5.2	28.4	5.8	*t* = −0.3 (df = 122)	0.781
Male	70	56.5	29	43.9	41	70.7	chi2(1) = 9.0	0.003
Female	54	43.6	37	56.1	17	29.3	-	-
**Education**								
< 12 years	46	37.1	16	24.2	30	51.7	chi2(1) = 10.0	0.002
≥ 12 years	78	62.9	50	75.8	28	48.3	-	-
**Diagnosis**								
Controls	33	26.6	29	43.9	4	6.9	chi2(3) = 40.6	< 0.001
SZP	35	28.2	18	27.3	17	29.3	-	-
BMD†	31	25.0	18	27.3	13	22.4	-	-
MPD	25	20.2	1	1.5	24	41.4	-	-
Total	124		66	53.2	58	46.8	-	-
**Substance**								
Alcohol			90	72.6	34	27.4	-	-
Cannabis			91	73.4	33	26.6	-	-
Meth			90	72.6	34	27.4	-	-
Other‡			113	91.1	11	8.8	-	-

SUD, substance use disorder; SZP, schizophrenia; MPD, methamphetamine induced psychotic disorder; s.d., standard deviation; BMD, body mass index; Meth, methamphetamine.

† , BMD includes bipolar I (*N* = 29) and bipolar II (*N* = 2).

‡ , Other’ includes methaqualone (*N* = 6), cocaine (*N* = 4), hallucinogens (*N* = 1), opioids (*N* = 1), inhalants (*N* = 0).

For patients with a major mood or psychotic disorder diagnosis, the duration of illness as reaching threshold for diagnosis was of a fairly long duration for most patients (BMD median = 72 months; SZP median = 72 months; MPD median = 12 months).

### Internal consistency of the Alcohol, Smoking and Substance Involvement Screening Test

Cronbach’s alpha for the TSI lifetime score (Q1–Q8) was 0.9, while the 3-month TSI was 0.8. We were unable to calculate Cronbach’s alpha for methaqualone, hallucinogens, opioids, and sedatives because of low prevalence. For the lifetime SSI scores, alcohol had a Cronbach’s alpha of 0.7, cannabis of 0.8, and methamphetamine of 0.9. Cronbach’s alpha for the SSI scores for the last 3 months were 0.6 for alcohol, 0.7 for cannabis, and 0.9 for methamphetamine, respectively.

### Discriminant validity of the Alcohol, Smoking and Substance Involvement Screening Test

Those with a SUD diagnosis on the SCID-I had significantly higher TSI scores compared to those without a SUD diagnosis (Table 2). The average lifetime TSI score in those diagnosed with a SUD was 31.3 with a standard deviation (s.d.) of 23.5, while for those without a SUD diagnosis it was 10.2 (s.d. = 9.4), which was a statistically significant difference (Table 2).

**TABLE 2:** Comparison of total substance involvement and specific substance involvement mean scores.

TSI/SSI	ASSIST score					
	SUD present	s.d.	SUD absent	s.d.	Stat	*P*
	Mean		Mean			
TSI lifetime†	31.3	23.5	10.2	9.4	*Z* = −6.4	< 0.001
TSI 3 months‡	22.7	18.8	5.2	7.4	*Z* = −4.2	< 0.001
SSI: Alcohol	6.9	5.3	3.7	6.0	*Z* = −4.2	< 0.001
SSI: Cannabis	6.7	7.5	1.4	4.4	*Z* = −4.9	< 0.001
SSI: Meth	10.0	10.4	0.1	0.4	*Z* = −9.1	< 0.001
SSI: Other§	8.8	11.0	0.5	1.7	*Z* = −5.3	< 0.001

ASSIST, Alcohol, Smoking and Substance Involvement Screening Test; SUD, substance use disorder; s.d., standard deviation; TSI, total substance involvement; SSI, specific substance involvement.

† , Total TSI score (sum of Q1–Q8).

‡ , TSI score for past 3 months only.

§ , ‘Other’ includes methaqualone, cocaine, hallucinogens, opioids, inhalants.

The 3-month mean TSI score in those diagnosed with a SUD was 22.7 (s.d. = 18.8), and for those without a SUD diagnosis it was 5.2 (s.d. = 7.4), which was also statistically significant. The SSI scores for alcohol, cannabis, methamphetamine, and other drugs were also significantly higher for those participants who had a SUD diagnosis.

The mean scores on the ASSIST TSI and SSI were then compared across the three categories of no-SUD, substance abuse, and substance dependence (Table 3). Bonferroni corrected post-hoc analysis for the TSI showed good discrimination between no-SUD and substance abuse (*p* < 0.001) and dependence (*P* < 0.001), respectively. However, discrimination between substance abuse and dependence was not significant (*P* = 0.564). This was also true for alcohol (no-SUD vs. abuse, *p* = 0.003; no-SUD vs. dependence, *p* < 0.001; abuse vs. dependence, *p* = 0.538). The ASSIST did not distinguish well between no-SUD and abuse for cannabis (*p* = 0.18), or between abuse and dependence (*p* = 0.533). However, it discriminated well between no-SUD and dependence (*p* < 0.001). Regarding methamphetamine, the ASSIST discriminated well between no-SUD and abuse (*p* = 0.003), no-SUD and dependence (*p* < 0.001), and between abuse and dependence (p = 0.039). For other drugs, the ASSIST discriminated between no-SUD and dependence (*p* < 0.001), however not between no-SUD and abuse (*p* = 0.189), or between abuse and dependence (*p* = 0.562).

**TABLE 3:** Comparison of total substance involvement and specific substance involvement mean scores across categories of no substance use disorder, substance abuse and substance dependence.

TSI/SSI	No SUD	Abuse		Dependence			Stat (ANOVA)	*P*
	Mean score	s.d.	Mean score	s.d.	Mean score	s.d.		
TSI total	10.2	9.4	28.8	22.4	34.3	25.0	Chi2 = 41.7	*p* < 0.001
							df = 2	
SSI alcohol	3.7	6.0	5.9	4.3	8.7	6.4	Chi2 = 17.3	*p* < 0.001
							df = 2	
SSI cannabis	1.4	4.4	4.0	5.9	7.3	7.8	Chi2 = 16.7	*p* < 0.001
							df = 2	
SSI meth	0.1	0.4	5.9	8.9	11.3	10.6	Chi2 = 48.3	*p* < 0.001
							df = 2	
SSI other†	0.5	1.7	5.0	7.1	9.7	11.8	Chi2 = 13.0	*p* = 0.002
							df = 2	

SUD, substance use disorder; TSI, total substance involvement; SSI, specific substance involvement; s.d., standard deviation; ANOVA, analysis of variance.

† , ‘Other’ includes methaqualone, cocaine, hallucinogens, opioids, inhalants.

### Receiver operating characteristic analysis

**FIGURE 1:**
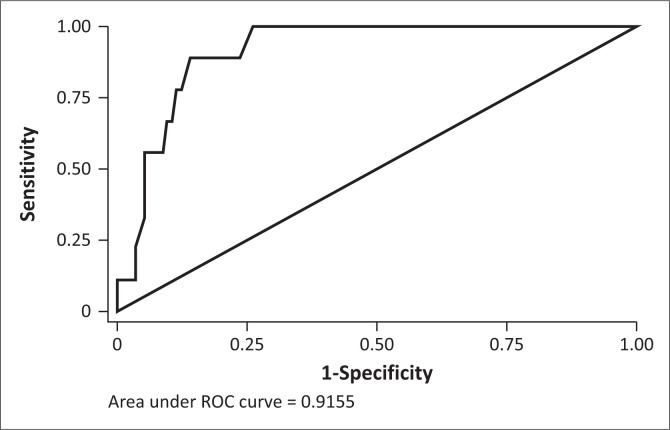
Receiver operating characteristic curve for the 3-month total substance involvement score.

**FIGURE 2:**
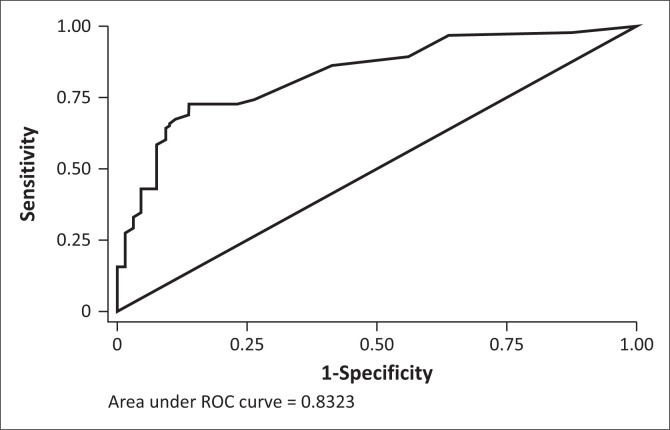
Receiver operating characteristic curve for the total substance involvement lifetime score.

We then determined the optimal cut-points for the TSI and SSI’s, with maximal balance between sensitivity and specificity (Table 4). It was found that a cut-off score of 13 on the TSI correctly classified 74.1% of cases and 74.2% of non-cases of SUD. The 3-month TSI score cut-off of 11 was found to correctly identify 88.9% of cases and 86.1% of non-cases. For alcohol, cannabis, methamphetamine, and ‘other drugs’, SSI lifetime cut-off scores of 4, 3, 3, and 3 respectively were found to have optimal balance between sensitivity and specificity. However, the cut-off scores for 3-month SSI scores for alcohol, cannabis, methamphetamine, and ‘other drugs’ were found to be higher, at 7, 10, 13, and 10 respectively. The area under the curve (AUC) was 0.7 or above for both the TSI (Figure 1 and Figure 2) and SSI’s.

**TABLE 4:** Cutoff points for total substance involvement and specific substance involvement scores.

TSI/SSI	Cut point	Sensitivity (%)	Specificity (%)	LR+	PPV	NPV	AUC
TSI (lifetime+3 months)	≥13	74.1	74.2	2.9	71.7	76.6	0.83
TSI (3 months)	≥11	88.9	86.1	6.4	88.8	86.1	0.92
SSI – alcohol (lifetime)	≥4	70.6	72.2	2.5	70.6	72.2	0.74
SSI – alcohol (past 3 months)	≥7	66.7	85.1	4.5	66.7	85.1	0.77
SSI – cannabis (lifetime)	≥3	60.6	85.7	4.2	60.6	85.7	0.74
SSI – cannabis (past 3 months)	≥10	100	96.7	30.5	100	96.7	0.98
SSI – meth (lifetime)	≥3	79.4	97.8	35.7	79.4	97.8	0.89
SSI – meth (past 3 months)	≥13	80.0	98.3	47.6	80.0	98.3	0.88
SSI – other (lifetime)	≥3	72.7	86.7	5.5	72.7	86.7	0.83
SSI – other† (past 3 months)	≥10	100	98.4	61.5	100	98.4	0.99

TSI, total substance involvement; SSI, specific substance involvement; LR+, positive likelihood ratio; PPV, positive predictive value; NPV, negative predictive value; AUC, area under the curve.

† , ‘Other’ includes methaqualone, cocaine, hallucinogens, opioids, inhalants.

### Logistic regression analysis

Univariate logistic regression analyses were then performed. A TSI of above 13 was found to be a significant predictor of the likelihood of any SUD, with individuals scoring at or above 13, having an 8.3-fold increase in the odds of having a SUD. When adjusted for sex, education level, and diagnosis (controls as reference vs. SZP, vs. BMD, vs. MPD), the OR was slightly lower at 6.5. This cut-off correctly classified 80.7% of cases. For the 3-month TSI score cut-off of 11, the OR was 49.5, with an adjusted OR of 66.8; 95.2% were correctly classified (Table 5).

**TABLE 5:** Adjusted and unadjusted odds ratios for the 3 month and lifetime total substance involvement cutoff scores.

	OR	95% CI^1^	OR (adjusted)^2^	95% CI^1^ (adjusted)^2^	*P*
TSI lifetime cutoff ≥ 13	8.3	3.7–18.5	6.5	2.2–19.1	< 0.001
TSI 3 months cutoff ≥ 11	49.5	5.8–422.8	66.8	5.6–794.0	< 0.001

1 CI: confidence interval.

2 adjusted for sex, education level, and diagnosis.

## Discussion

This is the first validation study to be conducted for individuals with multi-episode bipolar or psychotic disorders. Over half of the cases in this study were found to meet criteria for SUD, compared to only 6.9% of controls, consistent with research showing the higher rate of SUD in this particular group.^3^ This further emphasises the need for brief, easy-to-administer screening tests that are valid in this particular patient group. The TSI score (lifetime and 3 months) was found to have high levels of internal consistency (Cronbach’s alpha 0.9), as did the SSI lifetime score (Cronbach’s alpha 0.8). A score of over 0.75 is considered to indicate good reliability.^16^

The ASSIST TSI and SSI scores had high levels of discriminant validity, as they showed significant differences across SCID-I substance use diagnoses. Moreover, after adjustment for variables that differed on the presence or absence of SUDs, namely sex, education level, and diagnostic status (including non-psychotic controls), the TSI cut-scores were still associated with significantly greater odds for being classified as having a SUD. A TSI cutoff score of 13 had a positive predictive value (PPV) of 71.7, and a negative predictive value (NPV) of 76.6. Individuals with this score or over were 6.5 times more likely to have a SUD, when adjusted for sex, education, and diagnosis. The 3-month TSI score had an even higher PPV at 88.8, with an NPV of 86.1. Bonferroni post-hoc analysis showed that the ASSIST TSI can discriminate well between individuals who have no SUD, and those who have either substance abuse or dependence as per the DSM-IV-TR criteria. Altogether, the TSI discriminated well between no SUD and abuse or dependence. For the SSI’s, the discrimination between no substance use and either abuse or dependence was generally better than the discrimination between abuse and dependence. All the SSI scores were able to discriminate between no-SUD and dependence. However, it should be noted that the ASSIST was not able to discriminate well between substance abuse and dependence for all substances except for methamphetamine. These findings are in keeping with concerns about the substance abuse diagnosis in DSM-IV-TR, including the lack of a clear conceptual core, and a lack of empirical distinctions between substance abuse and dependence.^17^ The more recently published and current version of the DSM (DSM-5) no longer differentiates between substance abuse and dependence. Instead it classifies SUDs into mild (2–3 symptoms), moderate (4–5 symptoms), and severe (6 or more symptoms).^18^ Thus our finding of a lack of distinction between abuse and dependence is in keeping with the latest changes to the DSM.

Receiver Operating Characteristic analysis found a cutoff score for the lifetime TSI score to be 13. This is slightly lower than the score of 16 found by Hides et al.,^13^ 14.5 by Humeniuk et al.,^11^ 15 by Newcombe et al.,^8^ and 22 determined by Van Der Westhuizen et al.^12^ The AUC value was between 0.8 and 0.9, which is considered to be an excellent level of diagnostic accuracy.^19^ A score of over 13 would thus indicate a need for intervention or treatment for SUD.

The AUC was greater for the 3-month TSI score compared to the lifetime TSI score, reflecting greater accuracy. We believe that this may in be in part because of recall bias, with recall of recent events being more accurate.

Regarding the SSI scores, we found that cutoff scores of 4 for alcohol, and 3 for cannabis, methamphetamine and ‘other drugs’, were indicative of a SUD, thus indicating a need for further assessment and intervention in those scoring above these scores. This is generally in line with the WHO recommendations, which has a cutoff of 3 for a brief intervention for most drugs (except alcohol, which has a cutoff of 10). These scores are similar, if slightly higher, to those found by Hides et al. (who also determined an alcohol SSI cutoff of 4, 2 for cannabis, and 1 for amphetamine).^13^ In line with other research, including that by Van Der Westhuizen et al., the SSI score for alcohol is lower than that found in the WHO study.^12^

As outlined earlier, SUDs in the context of bipolar and psychotic illness pose significant challenges.^13,14,15^ Given the constraints on health care services in South Africa^20^ we surmise that providing regular screening in the outpatient setting may be challenging. A brief, validated screening test which facilitates early and appropriate interventions may thus potentially help to improve physical and mental health outcomes, general functioning, and psychosocial problems in this vulnerable patient population. Dual diagnosis services, which integrate mental health services and substance use interventions, are recognised as an evidence-based^21^ intervention for patients diagnosed with both a mental illness and a SUD. However it has been shown that basic substance interventions are seldom offered in mental health care settings.^21^ This suggests that access to services might be problematic, especially in the South African setting where resources for mental health are constrained.^20^ The group of patients scoring highly on the ASSIST and found to have a SUD would require comprehensive, holistic interventions including motivational and/or behavioural interventions, family interventions, assistance with housing, rehabilitation, and psychopharmacology.^22^ While it would likely be challenging to provide this in the context of constrained outpatient settings, it has been shown that outpatient substance interventions are useful in practice, reducing subsequent psychiatric hospitalisations.^23^

### Strengths and limitations

Limitations of this study include the exclusive use of clinical interviews (the SCID-I) to determine the presence or absence of a SUD, without any confirmatory biological measures of substance use. Other limitations include the sample size that was limited to that of the parent study, possibly leading to diminished power in some analyses. While we were able to perform analyses for alcohol, cannabis, and methamphetamine, the sample size for methaqualone, hallucinogens, opioids, and sedatives was too low to be able to analyse data for these substances independently. Given that alcohol and cannabis are among the most abused substances in South Africa,^24^ the ASSIST remains very relevant to local conditions despite this limitation.

The 3-month ASSIST score was compared to a 1-month (‘current’ SUD) measure on the SCID-I. Thus, the time frames were slightly different, possibly affecting the results relating to these measures. However, this would not apply to lifetime measures.

Although all participants were ambulatory, stable outpatients, this was a heterogenous group with differences in illness duration and illness severity that could have affected reporting of substance use. Future studies may need to consider the impact of variables such as psychosis severity and may benefit from fuller characterisation of psychotic symptom course. As this study is a secondary analysis, the sample and controls were chosen with different study aims in mind. This may affect the generalisability of these findings to the real-world clinical populations.

The strengths of the study include the fact that it is the first to examine the validity of the ASSIST in a population with multi-episode bipolar or psychotic illness, with a diverse range of diagnoses, as well as the inclusion of a control group without bipolar or psychotic illness. The patient sample included a diverse range of illness (SZP, bipolar and methamphetamine induced psychosis).

Further research is needed to address some of the limitations of this study, including validating the ASSIST in an inpatient setting and using a larger sample size especially for patients using methaqualone, hallucinogens, opioids and sedatives. A primary study rather than a secondary one may allow the findings to be more generalisable.

## Conclusion

The ASSIST is a brief, easy to use intervention which has validity in individuals with multi-episode bipolar or psychotic illnesses. It can be recommended as a tool to screen for SUDs in general, as well as specifically for alcohol, cannabis, and methamphetamine use disorders, in this population in order to identify those requiring further intervention and/or treatment.
